# *Spodoptera littoralis* oral secretions inhibit the activity of *Phaseolus lunatus* plasma membrane H^+^-ATPase

**DOI:** 10.1371/journal.pone.0202142

**Published:** 2018-08-10

**Authors:** Lorenzo Camoni, Francesca Barbero, Patrizia Aducci, Massimo E. Maffei

**Affiliations:** 1 Dept. Biology, Universtity of Rome Tor Vergata, Via della Ricerca Scientifica, Rome, Italy; 2 Dept. Life Sciences and Systems Biology, University of Turin, Turin, Italy; Natural Resources Canada, CANADA

## Abstract

Biotic stresses induced by herbivores result in diverse physiological changes in plants. In the interaction between the Lima bean (*Phaseolus lunatus*) and the herbivore *Spodoptera littoralis*, the earliest event induced by feeding on leaves is the depolarization of the plasma membrane potential (Vm), which is the results of both mechanical damage and insect oral secretions (OS). Although this herbivore-induced Vm depolarization depends on a calcium-dependent opening of potassium channels, the attacked leaf remains depolarized for an extended period, which cannot be explained by the sole action of potassium channels. Here we show that the plasma membrane H^+^-ATPase of *P*. *lunatus* leaves is strongly inhibited by *S*. *littoralis* OS. Inhibition of the H^+^-ATPase was also found in plasma membranes purified from leaf sections located distally from the application zone of OS, thus suggesting a long-distance transport of a signaling molecule(s). *S*. *littoralis’* OS did not influence the amount of the plasma membrane H^+^-ATPase, whereas the levels of membrane-bound 14-3-3 proteins were significantly decreased in membranes purified from treated leaves. Furthermore, OS strongly reduced the *in vitro* interaction between *P*. *lunatus* H^+^-ATPase and 14-3-3 proteins. The results of this work demonstrate that inhibition of the plasma membrane H^+^-ATPase is a key component of the *S*. *littoralis* OS mechanism leading to an enduring Vm depolarization in *P*. *lunatus* wounded leaves.

## Introduction

Plants use a sophisticated sensory system to detect potential enemies and subsequently to translate and integrate such signals into appropriate biochemical and physiological responses. In plant-insect interactions, herbivory combines two different sites of the feeding process: the mechanical wounding of the infested tissue and the introduction of oral secretions (OS). OS from feeding insects contain herbivore-specific compounds with elicitor-like properties [1]. Following herbivory and the release of OS, there is an increased accumulation of cytosolic calcium [Ca^2+^]_cyt_ and reactive oxygen species (ROS, e.g., H_2_O_2_) and NO release [2, 3]. Furthermore, changes of plasma membrane potentials (Vm), which are mainly caused by a calcium-dependent potassium channel activation [4] precede the cascade of events eventually leading to gene expression of defense responses [5–7].

Previous work has shown that the interaction between the Lima bean (*Phaseolus lunatus*) and the herbivore *Spodoptera littoralis* can be considered an ideal model to evaluate early and late responses of plants to herbivory [8–10]. The recent discovery of a putative β-galactofuranose polysaccharide in the OS of *S*. *littoralis* that is able to trigger early plant responses, and the finding that the responses to herbivory can be separated into a calcium-activated oxidative response and a K^+^-dependent Vm-activated jasmonate response associated with the release of volatile organic compounds (VOCs) [4], opens new questions on the role of the plasma membrane in early detection of biotic attacks. Moreover, upon herbivory, wounded leaves maintain Vm depolarized conditions that cannot be recovered by the activation of ion channels [2].

In plants, the plasma membrane H^+^-ATPase is responsible for the establishment of a proton electrochemical gradient across the plasma membrane that is utilized by channel and carrier proteins for the transport of ions and solutes [11]. Hence, variations in H^+^-ATPase activity can modulate membrane potential, thereby influencing the activities of voltage-gated channels and controlling ion flux at the plasma membrane [12].

In addition to its pivotal role in the regulation of basic aspects of plant cell function, H^+^-ATPase takes part in signaling events in response to diverse environmental stimuli, including pathogens [13]. The main regulatory mechanism of the plasma membrane’s H^+^-ATPase involves its interaction with 14-3-3 proteins, a family of eukaryotic regulatory proteins involved in the regulation of fundamental physiological processes in plants through phosphorylation-dependent interactions with client proteins [14, 15]. The association of 14-3-3 proteins with the C-terminal autoinhibitory domain of H^+^-ATPase brings about its displacement and consequent enzyme activation [16, 17]. The H^+^-ATPase binding site for 14-3-3s is generated upon phosphorylation of a conserved threonine residue within the sequence YTV, located at the very end of the C terminus [16]. To our knowledge, nothing is known on the effect of herbivores’ OS on the activity of plants’ H^+^-ATPase. Given the pivotal role of the plasma membrane H^+^-ATPase in the establishment and maintenance of the Vm, it is conceivable that this enzyme might be related to plant responses to insects’ OS.

The natural toxins fusicoccin (FC) and okadaic acid (OKA) induce a Vm hyperpolarization by H^+^-ATPase activation and therefore are useful tools to investigate the regulatory mechanisms of the proton pump. FC activates the H^+^-ATPase by irreversibly stabilizing the H^+^-ATPase/14-3-3 complex [16–21], while the protein phosphatase inhibitor OKA [22] activates the proton pump by blocking the dephosphorylation of its C-terminal domain and consequently promoting 14-3-3 binding [18].

In this work, we performed *in vivo* and *in vitro* studies to investigate the ability of *S*. *littoralis* to affect the activity of the Lima bean proton pump. Here we show that *S*. *littoralis*’ OS inhibit interaction between the H^+^-ATPase and 14-3-3 proteins in Lima bean leaves.

## Materials and methods

### Chemicals

Fusicoccin (FC) was prepared according to Ballio et al. [23]. Okadaic acid was purchased from Calbiochem, (La Jolla, California). [γ-32P]ATP (specific activity 110 TBq/mmol) was from Perkin Elmer (Boston, MA). Protein kinase A, catalytic subunit, and thrombin were from Sigma-Aldrich (St. Louis, Missouri). Chemicals for gel electrophoresis were from Bio-Rad (Hercules, California). All other reagents were of analytical grade.

### Plant and animal material

Lima bean (*Phaseolus lunatus* ‘Ferry Morse’ var. Jackson Wonder Bush) plants were grown in a growth chamber at 22 C, 80% humidity, under a 16 h light/8 h dark cycle for 2 weeks.

Eggs of *Spodoptera littoralis* Boisd. (Lepidoptera, Noctuidae) were kindly supplied by Syngenta (Switzerland) and larvae were fed on an artificial diet composed of 125 g bean flour, 2.25 g ascorbic acid, 2.25 g ethyl 4-hydroxybenzoate, 750 μl formaldehyde, 300 ml distilled water and 20 g agar previously dissolved in 300 ml distilled water. The ingredients (Sigma-Aldrich, St. Louis, MO, USA) were mixed with a blender and stored at 4°C for not more than 1 week. With the exception for VOC collection (see below), plants were fed for 2 h with third instar larvae reared from egg clutches in Petri dishes (6 cm diameter) in a growth chamber with 16 h photoperiod at 25°C and 60–70% humidity [24].

### Collection of oral secretions

*S*. *littoralis* oral secretions (OS) were obtained from 5-day-old larvae which were allowed to feed on Lima bean leaves for 24 h. Regurgitation was caused by gently squeezing the larva with forceps behind the head. OS were collected into glass capillaries connected to an evacuated sterile vial (peristaltic pump). Secretions were stored at -20°C until analysis. Five microliters of OS in 5 mM Mes-NaOH (pH 6.0) were applied to the leaf with a microsyringe and the Vm of leaves was analyzed after 2 h. The OS quantity was assessed after several trials (from 0.5 to 10 μl) and was found the most appropriate to obtain reproducible experiments.

### Plant treatments

Treatments were carried out by wounding the apex or primary leaves and then applying 10 μl OS or 10 μM FC to the apex or primary leaves. As a control, 10 μl deionized water was applied to wounded leaves without OS or FC application were used. For each treatment, at least three biological replicates were performed.

### Membrane potential determination

Membrane potentials were determined in leaf segments. The transmembrane potential (Vm) was determined using glass micropipettes with a tip resistance of 4–10 MΩ and filled with 3 M KCl, as previously described [8, 25]. Based on topographical and temporal determination of Vm performed previously, the electrode was inserted between 0.5 and 1.5 mm from the wounded zone, where a significant Vm depolarization occurs after HW. The results of all Vm measurements are shown as the average number of at least 50 Vm measurements.

### Plasma membrane purification

Plasma membranes were purified from Lima bean leaves following the procedure described by Serrano [26], with minor modifications. Twenty-five g of treated or control leaves were cut and homogenized with 25 ml of a buffer containing 25 mM MOPS-BTP, 250 mM sucrose, 5 mM EDTA, 2 mM DTT, 1 mM PMSF, 0.2% BSA, pH 7.8, filtered and centrifuged for 20 min at 8000 g at 4°C. The supernatant was then filtered and ultracentrifuged at 70000 g for 30 min. The pellet (microsomal fraction) was resuspended in 2 ml of 5 mM potassium phosphate buffer containing 0.2 mM PMSF, pH 7.8 and added to 14 ml of phase 7.2% Dextran T-500, 7.2% PEG-3350, 286 mM sucrose, 5.7 mM KCl, 5.7 K3PO4, pH 7.8. After mixing by repeated inversions, the samples were centrifuged at 2000 g for 15 min at 4°C. The upper phase, containing the plasma membrane fraction, was recovered, diluted twofold with the buffer 10 mM MOPS-BTP, 250 mM sucrose, 2 mM EDTA, 1 mM DTT, 1 mM PMSF, pH 7.0 and ultracentrifuged for 45 min at 125000 g at 4°C. The pellet was resuspended in 2 ml of GTED 20 buffer (10 mM Tris-HCl, 1 mM EDTA, 1 mM DTT, 20% glycerol, pH 7.6) and stored at -80°C.

### SDS-PAGE and Western blot

Immunodecoration of 14-3-3 and H^+^-ATPase was performed according to Muzi et al. [27]. In particular, plasma membrane proteins were separated by SDS-PAGE (Laemmli, 1970) using a mini-gel apparatus (Bio-Rad, Hercules, CA), then electroblotted onto a PVDF membrane with 39 mM glycine, 48 mM Tris, 0.1% SDS, 10% methanol. After blocking for 1 h in TTBS (20 mM Tris-HCl, pH 7.5, 100 mM NaCl, 0.05% Tween 20) with 5% no-fat dried milk at room temperature, the membrane was incubated with polyconal rabbit anti-14-3-3 antibodies recognizing all plant 14-3-3 isoforms or polyclonal anti-H^+^-ATPase antibodies directed against a conserved region in the C terminal domain. Following three washes with TTBS, the membrane was incubated with HRP-conjugated anti-rabbit secondary antibody (1:10000; Bio-Rad) and decorated with Clarity ECL-Western Blotting Kit (Bio-Rad).

### Overlay assay

The cDNA of the 14-3-3 isoform ω from Arabidopsis (14-3-3 PROTEIN G-BOX FACTOR14 OMEGA, AT1G78300), cloned into pGEX-2TK vector, was expressed in *Escherichia coli*, as previously described [28]. The expression system produces a GST-fused 14-3-3 containing a cAMP-dependent protein kinase phosphorylation site and a thrombin site between the two polypeptides. The ^32^P-labeled 14-3-3 ω was obtained as described by Pallucca et al. [28]. The specific activity of ^32^P-labeled 14-3-3 was 3.4 MBq/mg.

The overlay assay was performed according to Camoni et al. [29], with slight modifications. Two-phase partitioned plasma membranes (10 μg protein) were subjected to SDS-PAGE and blotted onto a nitrocellulose membrane, using a semidry apparatus (2 h, 0.8 mA cm^-2^). The membrane was blocked with 5% milk (fatty-acid–free) in 25 mM Hepes-OH, 5 mM MgCl2, 75 mM KCl, 1 mM DTT, 0.1 mM EDTA, 0.04% Tween-20, pH 7.5 (buffer H) and then incubated overnight at 4°C in the same buffer containing 3% fatty-acid–free milk and ^32^P-labeled 14-3-3ω (8.0 kBq/ml). The membrane was then washed extensively with buffer H, dried and subjected to autoradiography. The densitometric analysis was performed using ImageJ image-processing software [30]. The densitometric values are expressed as a percentage of the maximum integrated densitometric value (the product of the area and mean grey value).

### Protein content and H^+^-ATPase activity

Protein concentration was determined by the method set out by Bradford [31], using bovine serum albumin as a standard. H^+^-ATPase activity was assayed by measuring the release of inorganic phosphate, after Serrano [26]. In particular, 15 μg of plasma membrane vesicles from Lima bean leaves were incubated for 30 min in 500 μl of incubation buffer (50 mM Tris-MES, 5 mM MgSO4, 5 mM KNO3, 0.2 mM (NH_4_)_6_Mo_7_O_24_, pH 6.5) in the presence of 2 mM ATP. The reaction was stopped by the addition of 1 ml of phosphate reagent (0.5% SDS, 0.5% (NH_4_)_6_Mo_7_O_24_, 2% (v/v) H_2_SO_4_) and 0.01% ascorbate. The phosphate released during the reaction was determined by measuring the absorbance at 740 nm. The phosphate concentration was calculated by interpolation with a calibration curve obtained with different concentrations of potassium phosphate. For each sample, the residual activity in the presence of 0.2 mM of the H^+^-ATPase inhibitor ortovanadate was determined and subtracted from the obtained values for the calculation of H^+^-ATPase specific activity.

### Statistical analysis

The data given represent the mean of four independent experiments. Statistical significance was assessed by unpaired Student’s *t-*tests. All values are expressed as means ± s.e.m.

The minimal data set necessary to replicate the study findings is reported in S1 Dataset.

## Results

### The H^+^-ATPase activators fusicoccin and okadaic acid counteract the *Spodoptera littoralis* OS-induced plasma membrane depolarization in Lima bean leaves

The OS of *S*. *littoralis* induce a fast and persistent Vm depolarization of Lima bean leaves [8]. In order to investigate the possible role of the plasma membrane H^+^-ATPase on the Vm depolarization induced by *S*. *littoralis* OS, we evaluated the effect of OS perfusion on the Lima bean leaf Vm in the presence of either fusicoccin (FC) or okadaic acid (OKA). These natural toxins are known to induce a Vm hyperpolarization by H^+^-ATPase activation. The proton pump activation by FC is the consequence of the irreversible stabilization of the H^+^-ATPase/14-3-3 complex [16–19, 21], whereas the protein phosphatase 1-2A inhibitor OKA [22] activates the H^+^-ATPase by blocking the dephosphorylation of its C-terminal domain, consequently promoting 14-3-3 binding [18].

**Fig 1:**
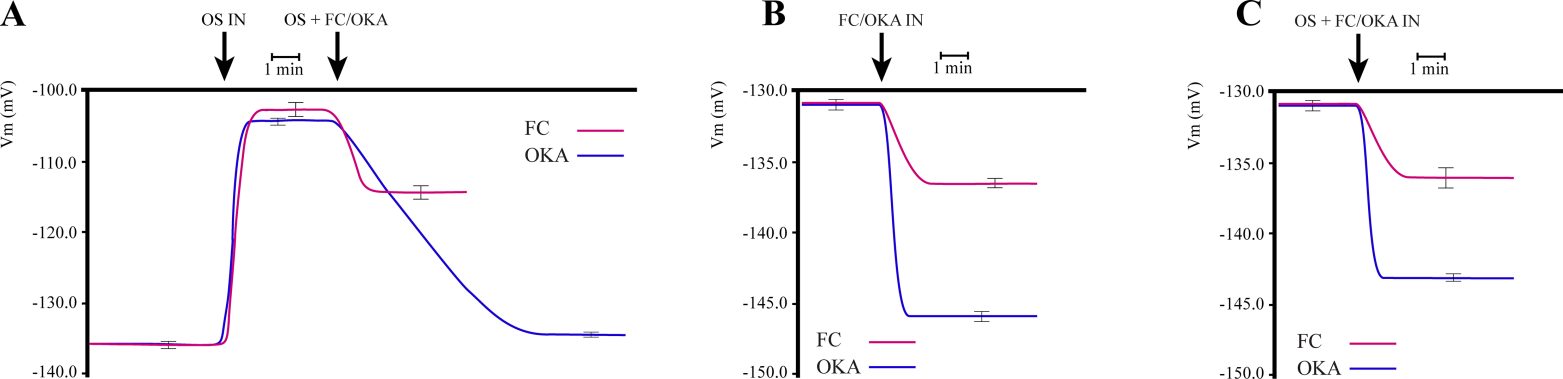
Effect of *Spodoptera littoralis* oral secretions (OS), okadaic acid (OKA) and fusicoccin (FC) on *Phaseolus lunatus* plasma membrane potential (Vm). **A**, Perfusion of OS causes a strong and significant (P < 0.05) Vm depolarization of *P*. *lunatus* mesophyll cell Vm, which is partly recovered by the addition of FC and totally restored to the initial Vm values by the addition of OKA. **B**, The perfusion of *P*. *lunatus* mesophyll cells with either FC or OKA causes a strong and significant (P < 0.05) Vm hyperpolarization, with a significantly higher OKA-dependent Vm hyperpolarization. **C**, The simultaneous perfusion of *P*. *lunatus* mesophyll cells with either OS + FC or OS + OKA shows inhibitory effects of the two toxins on the Vm depolarizing action of OS. Metric bars indicate standard deviation.

As expected, perfusion of *S*. *littoralis* OS induced a rapid and marked significant (P < 0.05) Vm depolarization in the Lima bean leaf, with respect to the starting conditions (Fig 1A). The addition of either FC or OKA to OS-depolarized leaves induced a significant (P < 0.05) Vm hyperpolarization, with respect to the OS-depolarized Vm value, by partially (FC) or fully (OKA) restoring the initial Vm potential (Fig 1A). When FC and OKA were perfused through Lima bean leaves, both drugs induced, to a different extent, a significant (P< 0.05) Vm hyperpolarization (Fig 1B), with respect to the starting condition. Intriguingly, the simultaneous administration of both of the inhibitors and *S*. *littoralis* OS partly reduced the OKA effect (Fig 1C), with respect to the sole administration of OKA (Fig 1B), whereas the combined effect of OS + FC (Fig 1C) was almost identical to the effect of the sole FC administration (Fig 1B). These data suggest that the proton pump could be a possible target of OS action.

### *Spodoptera littoralis* OS inhibit the activity of the Lima bean plasma membrane H^+^-ATPase

**Fig 2:**
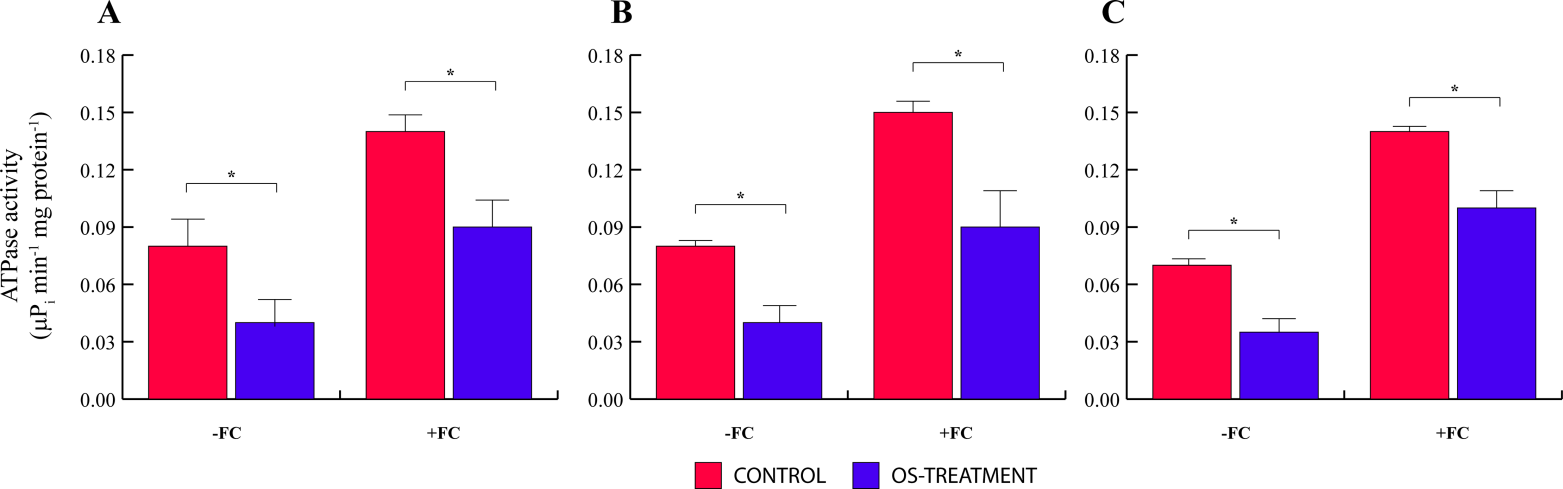
In vivo OS treatment inhibition of the of *P*. *lunatus* plasma membrane H^+^-ATPase activity. *A*pices of *P*. *lunatus* leaves were treated with either OS or FC and the vanadate-sensitive ATPase phosphohydrolitic activity was assayed as described in Materials and Methods. **A**. ATPase activity of whole leaf plasma membranes purified from controls and OS-treated plants without (-FC) and with the use of fusicoccin (+FC). **B**, ATPase activity of proximal half leaf plasma membranes purified from controls and OS-treated plants without (-FC) and with the use of fusicoccin (+FC). **C**, ATPase activity of plasma membranes purified from the distal half of the leaf plasma membranes purified from controls and OS-treated plants without (-FC) and with the use of fusicoccin (+FC). Data given are the mean of four independent experiments. Metric bars indicate standard deviation. Asterisk indicates significant (P< 0.05) differences.

In order to investigate whether the OS-induced Vm depolarization is also caused by the direct inhibition of the proton pump, we studied the *in vivo* effect of OS treatment on the phosphohydrolitic activity of the plasma membrane H^+^-ATPase. Lima bean leaves were treated with either OS or FC. Then plasma membranes were isolated by two-phase partitioning and the H^+^-ATPase activity was assayed *in vitro*. Treatment with OS consistently inhibited (-51%) the phosphohydrolitic activity of the H^+^-ATPase (Fig 2), with respect to the enzyme isolated from mock-treated leaves. When Lima bean leaves were simultaneously treated with OS and FC, the OS inhibitory effect was still present, albeit to a lower extent (-28%) (Fig 2).

Previous studies have demonstrated the existence of an OS-induced signal that travels through the phloem and induces the Vm depolarization of plant tissues in a systemic way [4]; therefore, we tested whether OS could induce H^+^-ATPase inhibition distantly from the site of OS application. To this purpose, Lima bean leaf apices were treated with OS or FC. After 5 h incubation, the leaves were cut in two parts, a proximal (towards the apex) and a distal (towards the petiole) portion, and the plasma membranes were extracted and purified. The plasma membrane H^+^-ATPase activity was similarly inhibited by OS both in the proximal and the distal half (Fig 2) of the Lima bean leaves.

### *Spodoptera littoralis* OS inhibits the association of 14-3-3 proteins with the Lima bean plasma membrane

**Fig 3:**
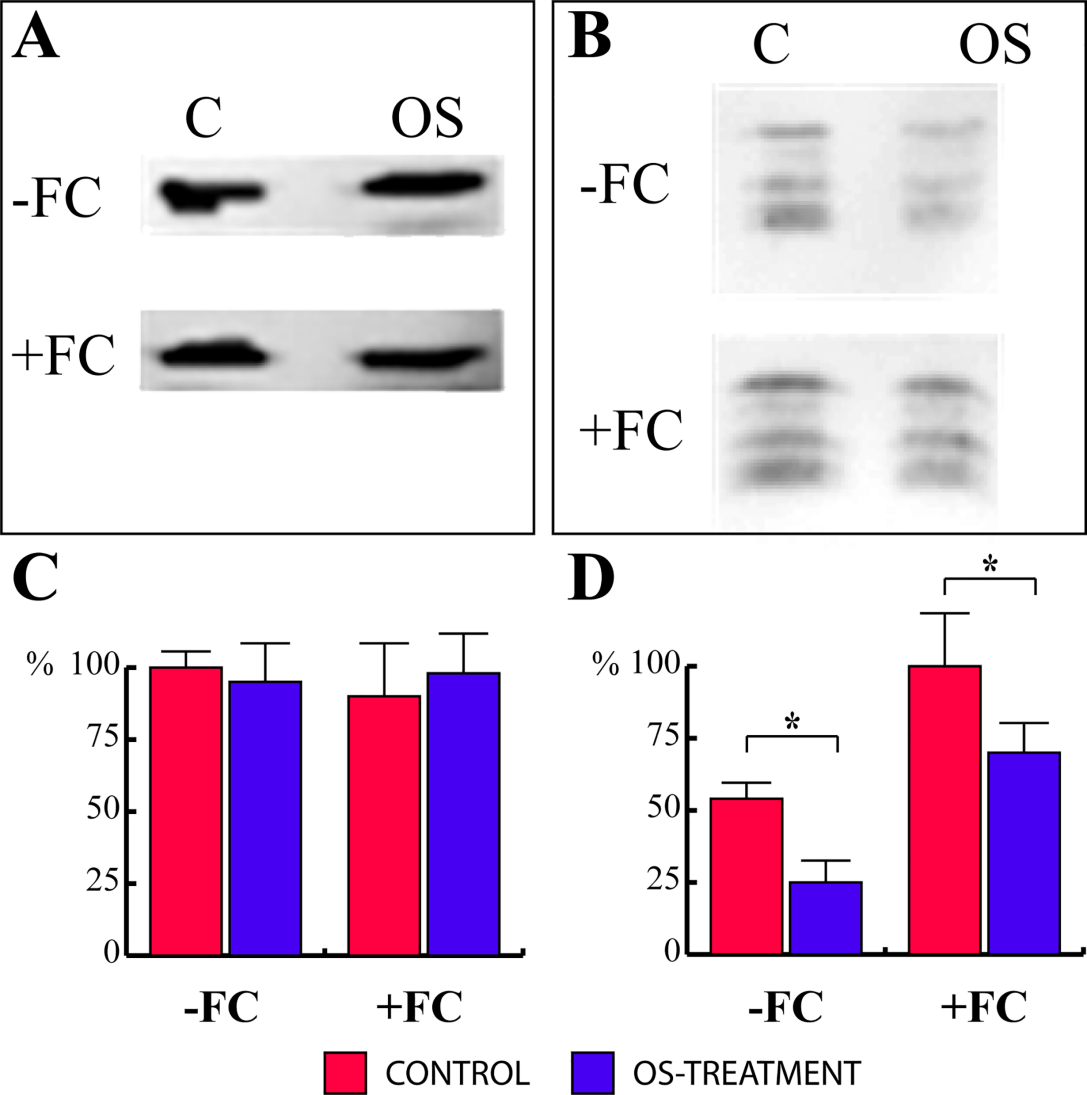
In vivo OS treatment inhibits the association of *P*. *lunatus* 14-3-3 proteins with the plasma membrane. **A**, SDS-PAGE analysis of plasma membranes of controls and OS-treated plants without (-FC) and with the use of fusicoccin (+FC) blotted onto nitrocellulose and probed with anti-H+-ATPase. **B**, SDS-PAGE analysis of plasma membranes of controls and OS-treated plants without (-FC) and with the use of fusicoccin (+FC) blotted onto nitrocellulose and probed with anti-14-3-3 antibodies. **C**, Densitometric analysis of H^+^-ATPase in controls (white bars) and OS-treated (black bars) plants without (-FC) and with the use of fusicoccin (+FC). D, Densitometric analysis of 14-3-3 proteins in controls and OS-treated plants without (-FC) and with the use of fusicoccin (+FC). Data are the mean of four independent experiments. Metric bars indicate standard deviation. C and D, expressed as percentage of maximum densitometric value. Asterisk indicates significant (P< 0.05) differences.

The inhibitory effect of OS on H^+^-ATPase activity can be potentially ascribed either to the inhibition of gene expression or to a post-translational mechanism involving the inhibition of 14-3-3 binding to the enzyme. We therefore analyzed H^+^-ATPase and 14-3-3 levels in the plasma membrane by western blot analysis in both control and OS-treated Lima bean leaves. The amount of H^+^-ATPase was not affected by the OS treatment either in the presence or absence of FC (Fig 3). On the other hand, the levels of membrane-bound 14-3-3 proteins were significantly decreased by the treatment with OS, also without the presence of FC (-50% by densitometric analysis, Fig 3). However, FC-treated leaves showed a significant (P < 0.05) increase of 14-3-3 levels when compared to leaves that were not treated with FC. Therefore, OS treatment, while ineffective on H^+^-ATPase content, was found to inhibit (-34%) association between 14-3-3 and the plasma membrane.

### *Spodoptera littoralis* OS inhibit the interaction between the H^+^-ATPase and 14-3-3 proteins in Lima bean

**Fig 4:**
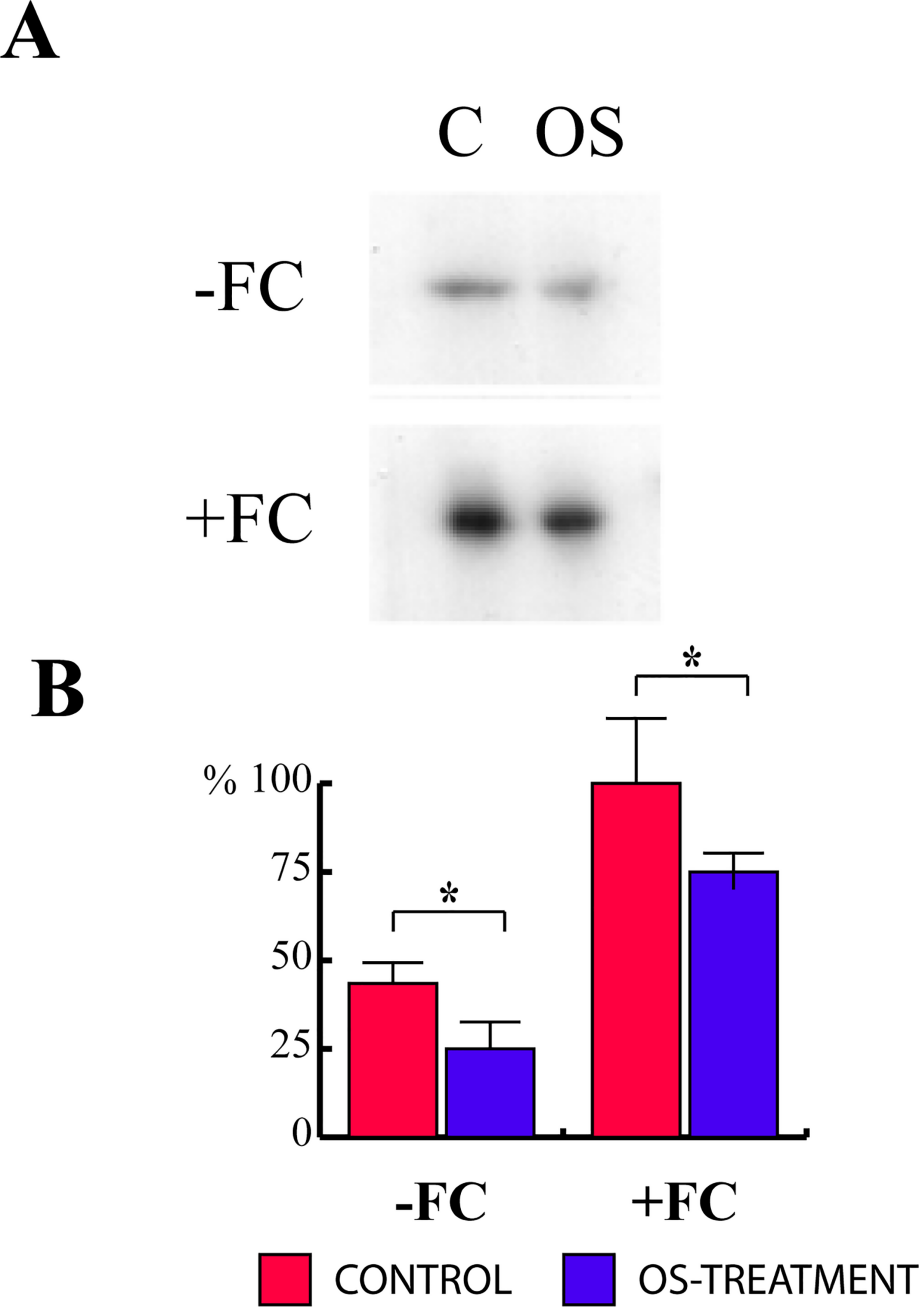
In vivo OS inhibition of the interaction between H^+^-ATPase and 14-3-3 proteins. **A**, Autoradiography and overlay assay performed using plasma membranes purified from control (C) and OS-treated *P*. *lunatus* leaves without (-FC) and after the addition (+FC) of 10 μM FC. **B**, Densitometric analysis of H^+^-ATPase controls (white bars) and OS-treated leaves (black bars) without (-FC) and with the use of fusicoccin (+FC). Data given are the mean of four independent experiments. Metric bars indicate standard deviation.

In order to verify whether OS were able to inhibit the association between H^+^-ATPase and 14-3-3 proteins, an *in vitro* overlay assay was performed. Plasma membranes from OS-treated Lima bean leaves were subjected to SDS–PAGE, blotted onto a nitrocellulose membrane and probed with ^32^P-labeled 14-3-3. OS treatment significantly (P < 0.05) reduced the association between the H^+^-ATPase and 14-3-3 proteins, both in the absence (-43%) and in the presence (-35%) of FC (Fig 4). These results suggest that the H^+^-ATPase might be a possible target of OS action.

## Discussion

The plasma membrane is the first line of defense against pathogens and herbivores. In plant-herbivore and plant-microorganism interactions, the plasma membrane responds with a Vm depolarization, depending on the nature of the biotic attack [32]. This Vm depolarization is associated with a Ca^2+^-dependent opening of K^+^ channels [4]; however, Vm depolarization has been found to persist for several hours after the application of OS following mechanical damage or after an herbivore attack [8, 9, 32]. Evidently, other factors besides K^+^ channel activity must be involved in order to sustain such a persistent Vm depolarization upon herbivory. For the first time, we provide evidence that the sustained Vm depolarization occurring after the administration of herbivore’s OS is associated with the inhibition of the plant plasma membrane H^+-^ATPase.

The first evidence of the direct involvement of H^+^-ATPase in response to the insect’s OS was the ability of FC or OKA to recover the membrane Vm depolarization, demonstrating that activation of the plasma membrane H^+^-ATPase can counteract the Vm depolarizing effects of OS. Interestingly, some differences between the OKA and FC effect could be observed. In fact, the protein phosphatase inhibitor OKA [18], which activates the H^+^-ATPase by blocking dephosphorylation of its C-terminal domain and consequently promoting 14-3-3 binding [19], completely restored the hyperpolarized Vm in OS-depolarized leaves, whereas FC was only able to partially suppress the OS effect. However, when OKA and OS were administrated simultaneously, the protein phosphatase inhibitor was not able to completely hamper the OS’ depolarizing effect. On the contrary, FC fully blocked the effects of the OS when administrated simultaneously. This effect can be ascribed to the ability of FC to irreversibly stabilize the H^+^-ATPase/14-3-3 complex [16–19] and suggests that the proton pump is involved in the OS-mediated Vm depolarization.

Upon herbivory, a wave of Vm depolarization spreads from the wounded zone to the rest of the leaf [8]. This event is associated with a hypothetical phloem mobile signal [2] that travels through active plasmodesmata [4]. The finding that the H^+^-ATPase activity of the plasma membrane was similarly inhibited by OS both in the proximal and the distal half of Lima bean leaves, indicates that OS can trigger the inhibition of the proton pump in intact (unwounded) tissues as well, as a result of a long-distance transport, in agreement with the hypothesis of a moving signaling molecule [2 and references cited therein].

It is well known that H^+^-ATPase is the main target of 14-3-3 proteins at the plasma membrane and its activation status is strictly correlated to membrane-bound 14-3-3 levels [18, 33]. As expected, 14-3-3 levels increased in plasma membranes purified from FC-treated leaves, since the toxin stabilizes 14-3-3 binding to the H^+^-ATPase [19]. It is well established that the H^+^-ATPase/14-3-3 interaction depends on the phosphorylation status of a Thr residue within a binding sequence located at the extreme end of the H^+^-ATPase C-terminus [16, 34]. Hence, the reduction in 14-3-3 proteins binding to H^+^-ATPase upon OS treatment could be ascribed to a Thr dephosphorylation. However, other mechanisms could be involved in the dissociation of 14-3-3 proteins induced by OS. In fact, it has been recently shown that the peptide hormone RALF and the flagellin fragment flg22 induce phosphorylation of Ser-899 in the H^+^-ATPase C terminus, which can inhibit the association of 14-3-3 proteins with the phosphorylated binding site Tyr-pThr-Val [11, 35, 36].

**Fig 5:**
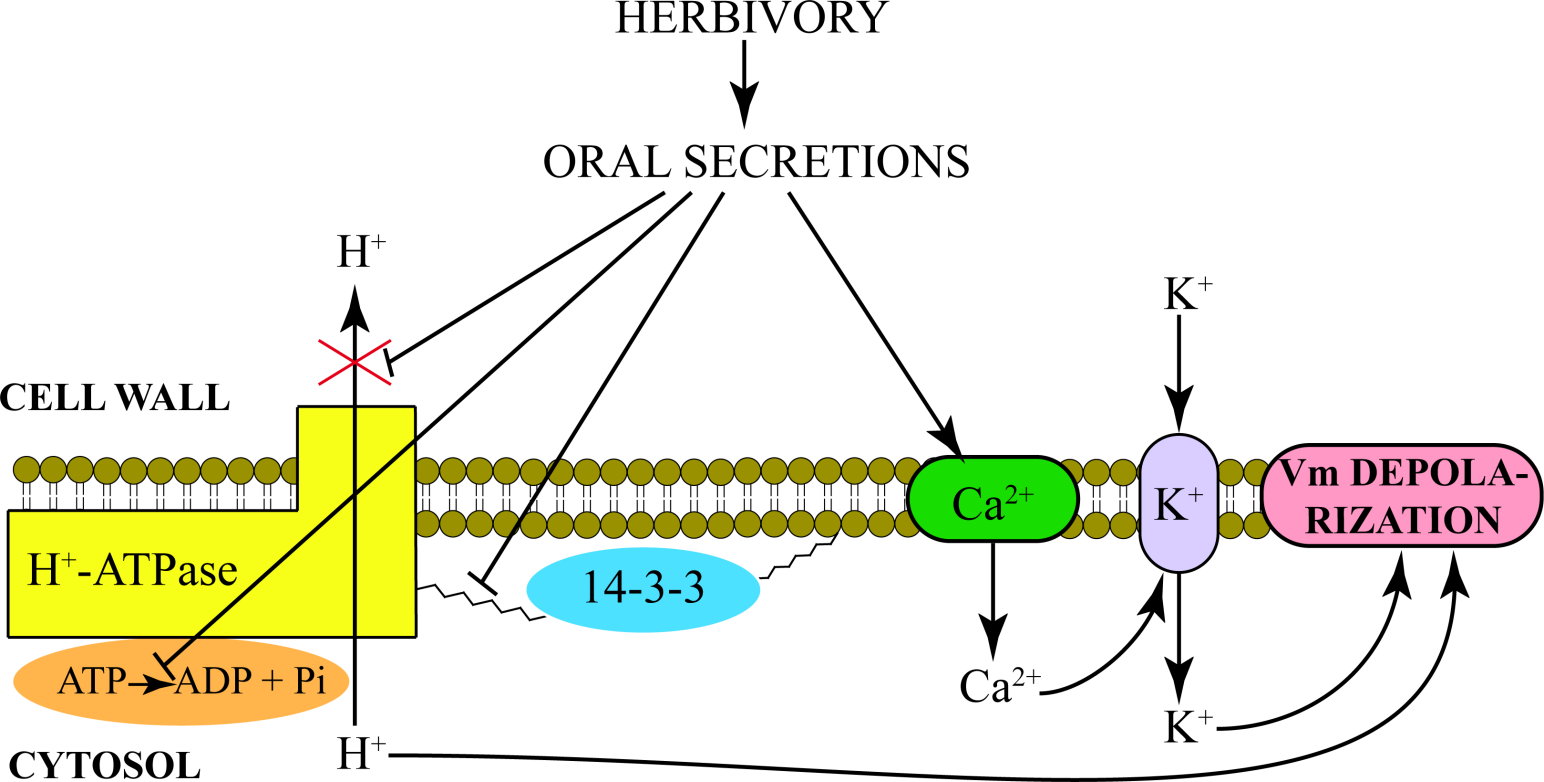
Early events upon *S*. *littoralis* herbivory on Lima bean leaves. Herbivory delivers oral secretions (OS) in wounded plant tissues that induce a calcium-dependent opening of K^+^ channels. The increased cytosolic K^+^ concentration causes a strong and sudden Vm depolarization. OS also inhibits the H^+^-ATPase activity by hampering the association between 14-3-3 and H^+^-ATPase proteins. The inhibitory action of OS causes a reduced phosphohydrolitic activity of the proton pump with a reduced extrusion of H^+^ from the cytosol. Overall, these events concur to the K^+^-dependent Vm depolarization.

To conclude, the present results, along with previous data, make it possible to better depict the early events taking place in a plant upon herbivory. Chewing insects such as *S*. *littoralis* release OS into wounded plant tissues and induce a calcium-dependent opening of K^+^ channels that causes a strong and sudden Vm depolarization. OS also inhibit the H^+^-ATPase activity by inhibiting the phosphorohydrolitic activity of the proton pump. Moreover, OS inhibit the association between 14-3-3 proteins and H^+^-ATPase, resulting in a reduced activity of the proton pump (with a reduced extrusion of H^+^ from the cytosol). It is likely that upon recognition of elicitors present in the OS the plant activates a signaling cascade that potentially de-phosphorylates the ATPase leading to its dissociation with the 14-3-3 proteins and reduced activity and subsequent effect on Vm. Overall, these events cause a Vm depolarization (Fig 5) that triggers late events that induce the typical plant response to herbivory [2].

Further studies will assess the immuno-purification of the ATPase from the plasma membranes and then the interaction assays on the purified protein. However, the overlay assay used in this study is recognized as an appropriate assay to study the H+-ATPase/14-3-3 interaction, by allowing to finely measure the affinity of the enzyme, which is strictly dependent to its phosphorylation status, towards a recombinant14-3-3 protein, as previously reported [16, 17, 19, 21].

## Supporting information

S1 Dataset (PDF)
