# Supplementary material for: Spodoptera littoralis oral secretions inhibit the activity of Phaseolus lunatus plasma membrane H+-ATPase
Source: PLoS One. 2018 Aug 10;13(8):e0202142. doi: 10.1371/journal.pone.0202142 (PMC6086434; doi:10.1371/journal.pone.0202142)
Supplement: S1 Dataset — (PDF) [file pone.0202142.s001.pdf]

Supporting Information.

Minimal data set necessary to replicate the study findings

Materials and Methods

Plant and animal material

Lima bean (*Phaseolus lunatus* ‘Ferry Morse’ var. Jackson Wonder Bush) plants were grown in a growth chamber at 22 C, 80% humidity, under a 16 h light/8 h dark cycle for 2 weeks.

Eggs of *Spodoptera littoralis* Boisd. (Lepidoptera, Noctuidae) were kindly supplied by Syngenta (Switzerland) and larvae were fed on an artificial diet composed of 125 g bean flour, 2.25 g ascorbic acid, 2.25 g ethyl 4-hydroxybenzoate, 750 µl formaldehyde, 300 ml distilled water and 20 g agar previously dissolved in 300 ml distilled water. The ingredients (Sigma-Aldrich, St. Louis, MO, USA) were mixed with a blender and stored at 4°C for not more than 1 week. With the exception for VOC collection (see below), plants were fed for 2 h with third instar larvae reared from egg clutches in Petri dishes (6 cm diameter) in a growth chamber with 16 h photoperiod at 25°C and 60-70 % humidity [24].

Collection of oral secretions

S. littoralis oral secretions (OS) were obtained from 5-day-old larvae which were allowed to feed on Lima bean leaves for 24 h. Regurgitation was caused by gently squeezing the larva with forceps behind the head. OS were collected into glass capillaries connected to an evacuated sterile vial (peristaltic pump). Secretions were stored at -20°C until analysis. Five microliters of OS in 5 mM Mes-NaOH (pH 6.0) were applied to the leaf with a microsyringe and the Vm of leaves was analyzed after 2 h. The OS quantity was assessed after several trials (from 0.5 to 10 µl) and was found the most appropriate to obtain reproducible experiments.

Plant treatments

Treatments were carried out by wounding the apex or primary leaves and then applying 10 µl OS or 10 µM FC to the apex or primary leaves. As a control, 10 µl deionized water was applied to wounded

leaves without OS or FC application were used. For each treatment, at least three biological replicates were performed.

Membrane potential determination

Membrane potentials were determined in leaf segments. The transmembrane potential (Vm) was determined using glass micropipettes with a tip resistance of 4–10 MΩ and filled with 3 M KCl, as previously described [8, 25]. Based on topographical and temporal determination of Vm performed previously, the electrode was inserted between 0.5 and 1.5 mm from the wounded zone, where a significant Vm depolarization occurs after HW. The results of all Vm measurements are shown as the average number of at least 50 Vm measurements.

Below is an example of a typical data matrix obtained

OKA2	OKA3	OS+FC1	OS+FC2	OS+FC3	OS+OKA1	OS+OKA2	OS+OKA3	OS---FC+OS1	OS---FC+OS2	OS---FC+OS3	OS---OKA+OS1	OS---OKA+OS2	OS---OKA+O[illegible]
-126.21369	-128.26651	-131.0008	-132.18127	-132.86196	-135.43977	-134.57103	-133.10124	-132.96998	-134.707883	-133.0544225	-134.5178038	-135.61064	-135.72629
-127.02602	-129.41497	-132.81232	-133.70828	-132.7211	-135.45659	-136.18287	-133.4974	-134.46739	-134.5061958	-133.1424586	-135.2296919	-137.0416284	-134.62425
-126.35134	-129.21889	-132.4898	-133.64425	-133.65826	-134.55382	-135.70109	-132.46659	-132.82353	-135.1484656	-132.1700684	-136.7699206	-133.9131594	-135.16847
-126.31291	-127.78552	-131.27091	-132.31532	-132.89236	-134.40096	-135.53262	-133.16047	-132.33694	-135.2084875	-133.2360975	-129.4870079	-135.9483749	-135.84554
-127.41258	-128.88916	-132.67427	-132.87516	-133.15326	-134.67827	-135.7495	-133.35575	-126.56942	-134.0756267	-133.3217344	-131.6586584	-136.4353746	-134.43858
-126.28171	-128.91076	-131.517	-133.70509	-133.66546	-133.35934	-134.30852	-132.52421	-131.76391	-135.212481	-132.030817	-130.2485019	-134.3633533	-134.10324
-126.97318	-128.07123	-131.43618	-132.60426	-131.91196	-135.31053	-135.45538	-132.89316	-135.72669	-135.3713572	-133.4970019	-137.4361813	-134.6618682	-135.7351
-127.60145	-128.2513	-132.56663	-131.73589	-132.95878	-134.17007	-135.29132	-132.34974	-133.66348	-134.3197376	-132.9927987	-132.9099685	-136.5890354	-134.64506
-125.7647	-128.95238	-131.59583	-133.73429	-133.14526	-134.75871	-134.7307	-131.66387	-134.34054	-135.0712329	-132.8679419	-135.3269368	-134.8527521	-134.63706
-125.23408	-127.09883	-132.84073	-132.31133	-132.84273	-135.2537	-134.92757	-132.86355	-134.82033	-134.191677	-132.9927987	-138.6478692	-134.3101263	-135.06123
-126.92517	-127.77592	-132.83555	-132.85594	-134.06083	-133.87196	-135.61624	-132.93317	-130.38015	-133.7582916	-132.1908779	-130.7134926	-136.663869	-134.51621
-125.89596	-129.00761	-131.32253	-133.39056	-134.12966	-134.88996	-134.49781	-132.41297	-135.04522	-134.9911988	-132.8919551	-133.2124919	-135.8139217	-134.34814
-126.42096	-128.64426	-132.69227	-132.07683	-132.75871	-133.61986	-136.07964	-133.77992	-133.05043	-133.891955	-132.7390916	-134.3961656	-134.3457401	-135.11885
-127.82593	-129.20448	-132.16686	-133.09044	-133.94079	-133.83994	-136.07924	-132.485	-132.99081	-134.8103285	-132.3589481	-131.672278	-136.8727535	-135.59264
-128.06962	-129.63505	-133.63185	-133.68587	-131.7407	-134.37815	-135.0092	-132.38336	-134.10245	-134.320125	-133.1760756	-130.6002438	-134.7551048	-135.13165
-126.5146	-128.27052	-132.39777	-131.60425	-132.41696	-133.39216	-135.82793	-132.73789	-135.76911	-133.6066574	-132.8903533	-136.9055659	-134.6062422	-133.80073
-127.45979	-127.80313	-132.08524	-132.82552	-133.18928	-134.91236	-134.58624	-133.39255	-134.57023	-135.3041232	-132.8359342	-130.8707595	-136.5370154	-134.69307
-128.11245	-128.7379	-133.27811	-132.17688	-132.36015	-134.37536	-135.02561	-132.57303	-134.89436	-134.140864	-133.9923984	-135.1908892	-134.9235773	-135.30052
-126.37095	-128.63706	-132.5138	-131.64906	-132.81313	-134.46538	-135.36334	-132.29612	-134.89436	-135.2384984	-132.7887124	-131.6666752	-136.1200511	-134.56663
-127.2677	-129.59903	-131.84273	-133.61624	-133.72388	-135.69549	-134.61666	-131.82152	-133.43057	-133.8971704	-132.5846336	-130.1864684	-136.9599849	-135.30133
-127.44817	-129.35574	-133.72109	-132.80512	-133.89996	-134.54582	-135.87755	-132.78831	-135.51861	-134.0740323	-133.4425754	-135.9315813	-134.5082074	-135.83193
-126.52302	-128.27852	-132.57703	-132.61625	-132.61625	-133.7359	-136.01762	-132.40496	-132.56782	-134.4117671	-132.6774753	-132.3753595	-135.4217678	-134.47059
-127.89476	-128.35374	-133.03881	-133.33614	-134.03682	-135.27091	-134.39776	-132.53782	-134.45377	-135.0340247	-132.1492589	-135.3165358	-137.2032911	-134.33053
-128.07323	-128.38495	-133.72789	-132.15646	-132.88675	-134.09564	-135.27532	-133.81513	-133.84634	-133.8731498	-133.8227264	-137.3561472	-135.2293044	-135.82793
-126.80674	-128.22249	-131.84794	-131.90357	-133.40536	-133.74509	-135.67387	-133.22369	-131.7607	-135.1220459	-132.4998013	-130.1312596	-136.4717931	-135.37176
-127.78752	-128.11524	-132.63466	-132.84594	-134.00081	-135.21929	-136.20128	-132.95798	-135.32172	-134.6574724	-132.9639874	-134.1680735	-137.1528506	-134.7347
-127.36376	-128.00641	-132.89316	-131.61545	-131.37816	-133.70588	-133.99881	-133.57823	-128.87475	-133.9147687	-132.8335351	-134.2713088	-136.9471848	-135.14446
-126.53382	-128.13847	-132.10444	-131.59944	-131.36454	-135.41536	-135.81152	-132.62184	-134.83395	-135.2441013	-131.9619886	-129.5018047	-136.0984445	-134.59544
-128.20768	-128.13766	-133.54742	-133.08443	-132.966	-133.43698	-136.4706	-132.71149	-131.02521	-135.0032091	-133.2737154	-136.4858001	-136.9259804	-134.55543
-127.7391	-128.01282	-133.60625	-132.67067	-131.64225	-133.65427	-134.62105	-132.17047	-130.45579	-134.2036724	-132.6566584	-134.6466541	-134.9715889	-135.34934
-127.37536	-129.01081	-132.2453	-132.27692	-133.83754	-134.90756	-134.7367	-132.9996	-134.69908	-134.8879486	-133.1496558	-133.2372874	-135.6890798	-134.92837
-128.54622	-128.35455	-133.4902	-132.9876	-134.18087	-133.47299	-135.489	-133.50541	-134.51461	-133.955583	-133.1864766	-137.1608675	-137.2545063	-135.57583
-128.36774	-128.80511	-132.2221	-131.7451	-133.40937	-134.91236	-134.62226	-133.11325	-134.91957	-133.9431852	-132.0572219	-134.4541907	-134.0408176	-134.72269
-126.68347	-129.60464	-131.7439	-131.84513	-134.16967	-134.95758	-135.37055	-132.59624	-134.37655	-134.9435747	-132.199677	-130.5034012	-135.3569478	-135.46619
-127.90556	-128.39536	-132.86395	-132.9864	-132.09444	-133.58824	-136.51422	-133.69428	-134.60265	-133.7379068	-132.7687151	-136.3937557	-136.683479	-135.05482
-128.27532	-128.70429	-132.93877	-131.91877	-132.966	-135.12646	-134.57583	-132.83394	-130.7543	-133.5590333	-132.2933233	-135.8191371	-135.3793442	-134.54181
-126.60745	-128.69789	-132.21488	-132.51981	-132.74829	-133.45578	-134.59624	-132.03042	-134.511	-134.2929155	-131.9771952	-131.3697547	-136.6038471	-135.83513
-127.92037	-128.08484	-132.72549	-133.30612	-132.55303	-134.21248	-135.62106	-133.48219	-135.62105	-133.9759976	-133.5522256	-130.7286918	-137.0600164	-135.33974
-126.91717	-128.78752	-132.85794	-132.14166	-133.81033	-134.34814	-134.87595	-133.45978	-134.89716	-135.0328177	-132.8407398	-131.0144067	-134.6582621	-133.97599
-126.72029	-128.82514	-131.79472	-132.89876	-132.70988	-133.70228	-135.49781	-132.53462	-135.80312	-133.7046772	-132.4477813	-128.4757853	-135.7002854	-135.06123
-128.52221	-128.02401	-133.9932	-133.29212	-131.61825	-134.27891	-136.08325	-133.96798	-133.62265	-134.0716183	-133.1952832	-130.6178421	-136.7875189	-135.58463
-127.66667	-128.91717	-133.15807	-131.14005	-132.95399	-134.30531	-134.50421	-133.65026	-134.11605	-135.1004392	-132.6814762	-135.7791126	-135.4241669	-134.40577
-127.65747	-128.81313	-132.90916	-132.30172	-133.91678	-133.42936	-135.06082	-133.65186	-134.16047	-133.8963658	-132.3613397	-132.2368979	-136.2945139	-135.57023

Plasma membrane purification

Plasma membranes were purified from Lima bean leaves following the procedure described by Serrano [26], with minor modifications. Twenty-five g of treated or control leaves were cut and homogenized with 25 ml of a buffer containing 25 mM MOPS-BTP, 250 mM sucrose, 5 mM EDTA, 2 mM DTT, 1 mM PMSF, 0.2% BSA, pH 7.8, filtered and centrifuged for 20 min at 8000 g at 4°C. The supernatant was then filtered and ultracentrifuged at 70000 g for 30 min. The pellet (microsomal

fraction) was resuspended in 2 ml of 5 mM potassium phosphate buffer containing 0.2 mM PMSF, pH 7.8 and added to 14 ml of phase 7.2% Dextran T-500, 7.2% PEG-3350, 286 mM saccarose, 5.7 mM KCl, 5.7 K3PO4, pH 7.8. After mixing by repeated inversions, the samples were centrifuged at 2000 g for 15 min at 4°C. The upper phase, containing the plasma membrane fraction, was recovered, diluted twofold with the buffer 10 mM MOPS-BTP, 250 mM sucrose, 2 mM EDTA, 1 mM DTT, 1 mM PMSF, pH 7.0 and ultracentrifuged for 45 min at 125000 g at 4°C. The pellet was resuspended in 2 ml of GTED 20 buffer (10 mM Tris-HCl, 1 mM EDTA, 1 mM DTT, 20% glycerol, pH 7.6) and stored at -80 °C.

SDS-PAGE and Western blot

Immunodecoration of 14-3-3 and H^+-ATPase was performed according to Muzi et al. [27]. In particular, plasma membrane proteins were separated by SDS-PAGE (Laemmli, 1970) using a mini-gel apparatus (Bio-Rad, Hercules, CA), then electroblotted onto a PVDF membrane with 39 mM glycine, 48 mM Tris, 0.1% SDS, 10% methanol. After blocking for 1 h in TTBS (20 mM Tris-HCl, pH 7.5, 100 mM NaCl, 0.05% Tween 20) with 5% no-fat dried milk at room temperature, the membrane was incubated with polyconal rabbit anti-14-3-3 antibodies recognizing all plant 14-3-3 isoforms or polyclonal anti-H^+-ATPase antibodies directed against a conserved region in the C terminal domain. Following three washes with TTBS, the membrane was incubated with HRP-conjugated anti-rabbit secondary antibody (1:10000; Bio-Rad) and decorated with Clarity ECL-Western Blotting Kit (Bio-Rad).

Below is an example of the results obtained from the densitometry of wb

densitometry wb14-3-3									mean
noFCnoOS	45	55	64	43	56	58	50	49	52.5
FC no OS	120	80	92	78	118	117	92	103	100
noFC+OS	17	35	36	17	21	33	34	15	26
FC+OS	54	75	70	83	84	58	59	53	67

densitometry wbATPase									
noFCnoOS	108	92	110	90	112	89	101	98	100
FC no OS	109	76	96	110	78	95	112	88	95.5
noFC+OS	110	72	100	78	102	110	88	92	94
FC+OS	115	70	92	122	114	90	89	88	97.5

Overlay assay

The cDNA of the 14-3-3 isoform ω from Arabidopsis (14-3-3 PROTEIN G-BOX FACTOR14 OMEGA, AT1G78300), cloned into pGEX-2TK vector, was expressed in *Escherichia coli*, as previously described [28]. The expression system produces a GST-fused 14-3-3 containing a cAMP-

dependent protein kinase phosphorylation site and a thrombin site between the two polypeptides. The ^{32}P-labeled 14-3-3 ω was obtained as described by Pallucca et al. [28]. The specific activity of ^{32}P-labeled 14-3-3 was 3.4 MBq/mg.

The overlay assay was performed according to Camoni et al. [29], with slight modifications. Two-phase partitioned plasma membranes (10 µg protein) were subjected to SDS-PAGE and blotted onto a nitrocellulose membrane, using a semidry apparatus (2 h, 0.8 mA cm^{-2}). The membrane was blocked with 5% milk (fatty-acid–free) in 25 mM Hepes-OH, 5 mM MgCl2, 75 mM KCl, 1 mM DTT, 0.1 mM EDTA, 0.04 % Tween-20, pH 7.5 (buffer H) and then incubated overnight at 4°C in the same buffer containing 3% fatty-acid–free milk and ^{32}P-labeled 14-3-3ω(8.0 kBq/ml). The membrane was then washed extensively with buffer H, dried and subjected to autoradiography. The densitometric analysis was performed using ImageJ image-processing software [30]. The densitometric values are expressed as a percentage of the maximum integrated densitometric value (the product of the area and mean grey value).

Below is an example of the results obtained from Overlay assays

overlay assay									
noFCnoOS	38	49	43	50	39	46	49	34	43.5
FC no OS	80	120	78	120	120	86	89	107	100
noFC+OS	17	32	27	33	29	20	25	13	24.5
FC+OS	55	75	58	65	50	70	79	68	65

Protein content and H^{+}-ATPase activity

Protein concentration was determined by the method set out by Bradford [31], using bovine serum albumin as a standard. H^{+}-ATPase activity was assayed by measuring the release of inorganic phosphate, after Serrano [26]. In particular, 15 µg of plasma membrane vesicles from Lima bean leaves were incubated for 30 min in 500 µl of incubation buffer (50 mM Tris-MES, 5 mM MgSO4, 5 mM KNO3, 0.2 mM (NH4)6Mo7O24, pH 6.5) in the presence of 2 mM ATP. The reaction was stopped by the addition of 1 ml of phosphate reagent (0.5% SDS, 0.5% (NH4)6Mo7O24, 2% (v/v) H2SO4) and 0.01% ascorbate. The phosphate released during the reaction was determined by measuring the absorbance at 740 nm. The phosphate concentration was calculated by interpolation with a calibration curve obtained with different concentrations of potassium phosphate. For each sample, the residual activity in the presence of 0.2 mM of the H^{+}-ATPase inhibitor ortovanadate was determined and subtracted from the obtained values for the calculation of H^{+}-ATPase specific activity.

Below are the results of ATPase activity

atpase activity distal leaf																	mean
no FC no O	0.086	0.082	0.084	0.064	0.073	0.063	0.064	0.077	0.078	0.07	0.076	0.069	0.072	0.072	0.073	0.0735	0.0735313
FC no OS	0.145	0.145	0.13	0.135	0.144	0.146	0.139	0.132	0.144	0.138	0.144	0.139	0.145	0.136	0.138	0.14	0.14
noFC+OS	0.056	0.046	0.056	0.02	0.032	0.035	0.056	0.03	0.038	0.038	0.041	0.035	0.037	0.045	0.037	0.038	0.04
FC+OS	0.098	0.085	0.099	0.11	0.087	0.088	0.123	0.11	0.095	0.099	0.112	0.087	0.11	0.093	0.088	0.1	0.099

atpase activity whole leaf																	
noFCnoOS	0.065	0.1	0.08	0.08	0.082	0.067	0.068	0.123	0.095	0.11	0.075	0.069	0.067	0.069	0.08	0.09	0.0825
FC no OS	0.155	0.13	0.145	0.111	0.123	0.152	0.144	0.145	0.157	0.158	0.143	0.144	0.148	0.135	0.144	0.146	0.1425
noFC+OS	0.025	0.068	0.031	0.075	0.032	0.042	0.038	0.042	0.065	0.025	0.038	0.04	0.039	0.029	0.038	0.037	0.0415
FC+OS	0.07	0.11	0.09	0.07	0.12	0.08	0.12	0.08	0.09	0.06	0.112	0.114	0.09	0.08	0.07	0.09	0.090375

atpase activity proximal leaf																	
noFCnoOS	0.08	0.088	0.09	0.075	0.083	0.083	0.088	0.09	0.075	0.083	0.083	0.087	0.082	0.091	0.084	0.082	0.084
FC no OS	0.16	0.138	0.148	0.152	0.139	0.145	0.152	0.155	0.13	0.153	0.155	0.161	0.13	0.151	0.159	0.156	0.149
noFC+OS	0.033	0.06	0.04	0.074	0.041	0.05	0.0489	0.051	0.039	0.045	0.042	0.039	0.058	0.061	0.032	0.03	0.0464938
FC+OS	0.118	0.07	0.073	0.099	0.078	0.109	0.121	0.128	0.085	0.078	0.076	0.128	0.098	0.088	0.088	0.068	0.0940625
